# Cryopreservation of Spin-Dried Mammalian Cells

**DOI:** 10.1371/journal.pone.0024916

**Published:** 2011-09-22

**Authors:** Nilay Chakraborty, Michael A. Menze, Jason Malsam, Alptekin Aksan, Steven C. Hand, Mehmet Toner

**Affiliations:** 1 Center for Engineering in Medicine and BioMEMS Resource Center, Massachusetts General Hospital, Harvard Medical School and Shriners Hospital for Children, Boston, Massachusetts, United States of America; 2 Department of Biological Sciences, Eastern Illinois University, Charleston, Illinois, United States of America; 3 Biostabilization Laboratory, Department of Mechanical Engineering, University of Minnesota, Minneapolis, Minnesota, United States of America; 4 Division of Cellular, Developmental, and Integrative Biology, Department of Biological Sciences, Louisiana State University, Baton Rouge, Louisiana, United States of America; University of California at Berkeley, United States of America

## Abstract

This study reports an alternative approach to achieve vitrification where cells are pre-desiccated prior to cooling to cryogenic temperatures for storage. Chinese Hamster Ovary (CHO) cells suspended in a trehalose solution were rapidly and uniformly desiccated to a low moisture content (<0.12 g of water per g of dry weight) using a spin-drying technique. Trehalose was also introduced into the cells using a high-capacity trehalose transporter (TRET1). Fourier Transform Infrared Spectroscopy (FTIR) was used to examine the uniformity of water concentration distribution in the spin-dried samples. 62% of the cells were shown to survive spin-drying in the presence of trehalose following immediate rehydration. The spin-dried samples were stored in liquid nitrogen (LN_2_) at a vitrified state. It was shown that following re-warming to room temperature and re-hydration with a fully complemented cell culture medium, 51% of the spin-dried and vitrified cells survived and demonstrated normal growth characteristics. Spin-drying is a novel strategy that can be used to improve cryopreservation outcome by promoting rapid vitrification.

## Introduction

Vitrification is the direct transition from a liquid to an ice-free glassy state upon cooling. This technique avoids the damaging effects of ice crystals, which are known to form during conventional cryopreservation with slow cooling. However, a major bottleneck of the vitrification technique is that it requires high concentrations of cryoprotectants (CPAs) to avoid ice-nucleation during cooling. Such high concentrations (6–8M) of CPAs are toxic to the cells [1] and, as a result, multiple steps and elaborate protocols are required to load and unload CPAs into cells. This makes vitrification a complex and difficult process. We developed an alternative approach to achieve vitrification without the need to incubate the cells in exceedingly high concentrations of CPA. The spin-drying technique was used to rapidly reach uniformly low moisture content (<0.12 gH_2_O/gdw) across the sample (<1 min), and a 1.8 M trehalose- was as CPA. The technique of spin-drying has previously been used by Chakraborty et al. [2] to create ultra-thin films of trehalose.

It has been established that in order to achieve vitrification at lower CPA concentrations ultra-fast heat transfer rates are required [1], [3]. Heat transfer rates can be increased by reducing the sample volume and increasing the cooling rate. A number of techniques have been used to increase the cooling rate by reducing sample volume, specifically for preservation of oocyte and other germ cells. Thin straws as well as have been used to minimize the volume to be vitrified [4], [5]. More recently, taking advantage of the high thermal conductivity and the small diameter of quartz crystal (QC) capillaries, mammalian cells have been vitrified using lower concentrations of CPAs using ultra-rapid cooling rates [6]. An alternative approach to reduce sample size can be creation of ultra-thin film using spin-drying, which will promote faster cooling rates for vitrification.

One approach to induce/facilitate vitrification is the reduction of the moisture content in the sample using desiccation prior to cryopreservation. Li et al. [7] studied storage of mouse spermatozoa at cryogenic temperatures following partial desiccation of the sample using evaporative drying in sessile droplets. Mouse spermatozoa samples were desiccated to an estimated moisture content of 0.26 gH_2_O/gdw. Although offspring was obtained by intra-cytoplasmic injection (ICSE) of dried-frozen sperm into oocytes, the viability of the spermatozoa was not preserved. Nevertheless, the treatment was enough to preserve the genetic material (i.e., nucleus) of the sperm. This study highlighted the benefits of pre-desiccating cells before cooling to cryogenic temperatures.

The most common approach to desiccating cells involves drying sessile droplets containing cells [8], [9], [10]. However, desiccation using evaporative drying of sessile droplets is inherently slow and non-uniform in nature [11]. A glassy skin forms at the liquid/vapor interface of the sample when the cells are desiccated in glass-forming solutions that contain lyoprotectants such as trehalose. This glassy skin slows and ultimately prevents further desiccation of the sample beyond a certain level of dryness and induces significant spatial non-uniformity of the water content across the sample [12], [13]. As a result, cells trapped in the partially desiccated sample underneath the glassy skin may not vitrify but degrade due to high molecular mobility. The development of a fast drying technique to achieve very low and uniform final moisture levels across the sample might overcome some of the shortcomings of the evaporative drying techniques. One such technique might be the recently developed technique of spin-drying [2]. Numerical and experimental analyses of this technique showed that forced convective removal of water from the sample by centrifugal force leads to rapid desiccation to a thin layer within which cells are embedded. Using spin-drying, cells can be desiccated at a significantly faster rate than most other techniques. This minimizes the time of exposure of cells to deleterious effects of high CPA concentrations.

In this study, we used spin-drying to rapidly and uniformly desiccate Chinese Hamster Ovary (CHO) cells to a very low moisture level before cooling to cryogenic temperatures. We used FTIR spectroscopy to demonstrate that cell suspensions that contain 1.8 M trehalose can be rapidly and uniformly desiccated to an average glass transition temperature (T_g_) of ∼4.43°C. We also used TRET1 trehalose transporters to introduce trehalose into the cells prior to desiccation [14]. Cells that were spin-dried and stored at cryogenic temperature maintained membrane integrity as high as 51% upon rewarming and rehydration. These cells showed similar growth characteristics as control cells. This study outlines a technique to cryopreserve mammalian cells using pre-desiccation by spin-drying. This technique can be used to improve the cryopreservation outcome of the conventional vitrification technique.

## Materials and Methods

### Cell Culture

Chinese hamster ovary (CHO) cells were obtained from American Type Culture Collection (ATCC, Manassas, VA), and cultured in Dulbecco' modified Eagle's medium (DMEM) (Invitrogen, Carlsbad, CA) supplemented with 10% fetal bovine serum (Atlanta Biologicals, Norcross, GA) and 2% penicillin-streptomycin (10 U/mL penicillin G and 10 µg/mL streptomycin sulfate, Invitrogen, Carlsbad, CA). Cultures were maintained in 25-cm^2^ T-flasks (Corning Incorporated, NY) at 37°C and equilibrated with 10% CO_2_–90% air. CHO cells transfected with the TRET1 trehalose transporter were cultured under similar conditions.

### Transfection of CHO cells with Trehalose Transporter

TRET1 trehalose transporter was expressed in CHO cells using transfection techniques described by Kikawada et al. (2007). The TRET1 expression vector (pPvTRET1-IRES2-AcGFP1) was kindly provided by Dr. Takahiro Kikawada at National Institute of Agrobiological Sciences, Ibaraki, Japan. The transporter sequence was cloned into a retroviral vector with a neomycin drug resistance gene pLNCX2 (Clontech, CA) for the stable transfection of wild-type CHO cells. The transfected cells were selected with 400 µM Geneticin (G418) for several weeks until colonies of drug resistant cells appear in the culture dishes. These colonies were expanded for generation of stably transfected CHO-TRET1 cells. CHO-TRET1 cells appeared morphologically similar to CHO cells following stable transfection (data not shown).

### Trehalose Loading and Quantification

High purity, low endotoxin trehalose dihydrate was obtained from Ferro Pfanstiehl (Cleveland, OH). Wild-type CHO cells and those expressing the TRET1 transporter were incubated for 4 h at 37°C in a fully-complemented medium containing 0.4 M trehalose. This incubation condition was found optimal for loading the maximum amount of trehalose into these cells (data not shown). Intracellular trehalose was quantified by high performance liquid chromatography (HPLC). After loading trehalose, cells were detached from flasks by trypsinization. Suspended cells were centrifuged, washed three times with PBS, and then lysed by freeze-thawing in ultra-high purity water (electrical resistance >18 mOhm). The washing procedure may lead to loss of some of the trehalose since the TRET1 acts as facilitated diffusion transporter. Intracellular trehalose concentrations were likely higher before the washing process and our values are conservative estimates of the intracellular trehalose. The solution containing the lysed cells was centrifuged at 16,000 *g* and the supernatant was retained for HPLC analysis. Quantification of the intracellular trehalose was performed using a Dionex HPLC system with a GP-50 gradient pump, an ECD50 electrochemical detector and a PA10 column (Dionex, Sunnyvale, CA). An AS50 auto-sampler and thermal compartment was used for sample handling and injection during analysis. Sample peaks were identified by comparison of retention times to standards, and calibration curves were linear over the range assayed (r^2^ = 0.92).

### Spin Drying

**Figure 1:**
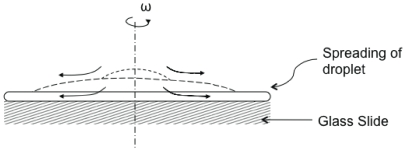
Basic configuration of the spin-drying apparatus. The cells were grown on glass cover slips prior to the spin-drying. During spin-drying, the glass cover slip was held in place by a vacuum chuck.

Spin-drying was performed using a commercially available spinning machine purchased from Brewer Science Inc. (Model Cee 200, St. Louis, MO). At about 80% confluence, the CHO cells were trypsinized with 0.25% trypsin/1 mM EDTA solution and placed in a tissue culture dish containing 22 mm round glass coverslips (BD BioCoat, San Jose, CA). The cells were incubated in cell culture medium and allowed to attach to the glass coverslips overnight (cf. [2]). To prevent cells from attaching to the edge of the coverslips, 20 mm diameter Press-to-Seal™ silicone isolators (Invitrogen, Carlsbad, CA) were used. Before spin-drying, the cell culture media was completely removed using a Pasteur pipette and immediately replaced with the drying solution (1.8 M trehalose, 10 mM KCl, 5 mM glucose, 20 mM HEPES, and 120 mM choline chloride at pH 7.4). Figure 1 shows the basic configuration of the spin-drying experiment. The spinning machine was equipped with a 1.5 inch vacuum chuck to hold the glass coverslips securely during spinning. The spinning compartment was fitted to a continuous purge of dry nitrogen gas supplied at 10 psi. Before spinning, the system was flushed with dry nitrogen gas. The glass coverslips were spun at 1000 rpm for 1 min. After drying, the samples were quickly removed from the spin-dryer and placed into a 35 mm cell-culture plate (MatTek Corp. Ashland, MA). Cells were rehydrated in 0.5 mL cell culture medium without phenol red. After recovery at 37°C and 10% CO_2_/90% air for 45 min, the membrane integrity of cells was determined by red-green fluorescence staining with Syto-13/ethydium bromide dyes.

### Glass Transition Temperature (T_g_) Measurements

Glass transition temperature (T_g_) of the spin-dried drying solution (composition: see above) was determined using temperature ramp FTIR spectroscopy [15]. FTIR measurements were performed using a Nicolet Continuum FTIR Spectromicroscope (Thermo Electron Corporation LLC, Waltham, MA) equipped with an MCT micron detector. IR spectra in the range of 930–8000 cm^−1^ were recorded using an aperture size corresponding to an area of 100×100 µm^2^ on the sample. The spectral resolution was 4 cm^−1^ and 128 co-added interferograms were averaged for each data point. A freeze-drying cryostage (FDCS 196, Linkam Scientific Instruments Ltd., UK) was mounted on the FTIR microscope for cooling/heating the samples at a controlled rate (2°C/min). FTIR samples were spin-dried on a CaF_2_ window. The samples were then sealed by vacuum grease and sandwiched between two CaF_2_ windows. To determine T_g_, the variation of the peak position of the ν-OH (3700–3000 cm^−1^) band was plotted against temperature [16].

### Quantification of Residual Water

The average moisture content of the samples was estimated by interpolation using the trehalose-water binary phase diagram [17] from the T_g_ measured using FTIR spectroscopy. Also, bulk gravimetric analysis of the water content of the spin-dried samples was performed using a high precision analytical balance (Metler Toledo XP Ultra Microbalance, Columbus, OH). The initial and final sample weights were measured and used to calculate moisture content. Dry weights of samples were determined by baking in a vacuum oven at a temperature below the glass transition temperature of trehalose (∼90°C) for 8 h.

### Storage Studies

Following spin-drying cell samples were immediately submerged in liquid nitrogen and stored for 1h at −196°C. To investigate the effect of slow freezing on viability of spin-dried samples, cells were cooled to −120°C at 1°C/min using a controlled rate freezer (Freeze Control® Cryopreservation System, Cryologic, Victoria, Australia) before storage in LN_2_ for 1 h. After storage, the cell-covered coverslip was quickly placed into a 35 mm glass-bottom cell-culture plate (MatTek Corp. Ashland, MA) and rehydrated in 0.5 mL cell culture medium without phenol red at 37°C for 45 min. Membrane integrity of the samples was measured by Syto-13/ethydium bromide membrane integrity assays as described above.

### Viability Assessment

The membrane integrity was determined using Syto-13/ethydium bromide membrane integrity assays (Molecular Probes, Eugene, OR). The stock solution for the Syto-13/ethydium bromide staining was prepared by adding 10 µL of 1 mg/mL Syto-13 solution (aq.) and 5 µL of 1.0 mg/mL solution ethydium bromide solution (aq.) to 8 mL of DMEM without phenol red or serum (Invitrogen Inc., Carlsbad, CA). After rehydration, 500 µL of Syto-13/ethydium bromide solution were added to the cells attached on coverslips, and the samples were incubated at 37°C for 5 min. These samples were then imaged using an inverted microscope (Carl Zeiss Biosystems, Oberkochen, Germany) using FITC and PI filters. Cell viability was determined immediately after rehydration with this technique by counting the live (green) and dead (red) cells in seven representative images taken at different locations on the coverslip. Long term viability and growth pattern of spin-dried cells was determined by incubating the rehydrated cell samples in fully complemented medium for 7 days in 35 mm cell culture dishes (Corning, Lowell, MA). In parallel samples, the viability of cells post-dehydration was measured on days 1, 3 and 7. To ensure that only viable cells were counted, the membrane integrity of these cells was determined by trypan blue exclusion. Phase contrast micrographs of cells were also collected at 10 random locations across the culture dish.

## Results

### Moisture Content and Glass Transition Temperature

**Figure 2:**
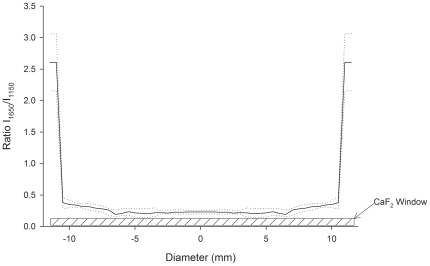
FTIR analysis of the dried film obtained after spin drying of sample buffer (1.8 M trehalose, 10 mM KCl, 5 mM glucose, 20 mM HEPES, 120 mM choline chloride, pH 7.4). The ratio of I_1650_/I_1150_ was used to quantify local water contents.

**Figure 3:**
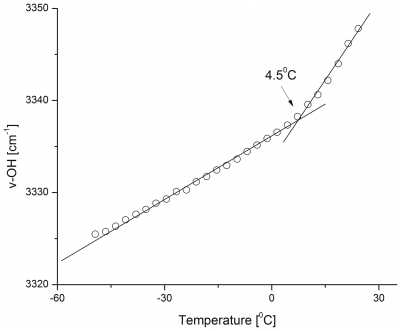
Glass transition temperature of the dried spinning solution (1.8 M trehalose, 10 mM KCl, 5 mM glucose, 20 mM HEPES, 120 mM choline chloride, pH 7.4) using FTIR technique. The figure indicates a characteristic peak-position of ν-OH stretch plotted against decreasing temperature.

Spin-dried samples were analyzed to determine the spatial variation of water content by FTIR spectroscopy (Figure 2). The small variation in the ν-OH peak ratio across the specimen indicated the high spatial uniformity of the desiccated sample. It is important to note that water content at the edge of the spin-dried sample was the highest. This ‘edge effect’ was highly localized (Figure 2) and is an artifact of the spin-drying technique, likely caused by surface tension [18]. Temperature-ramp FTIR spectroscopy indicated that spin-dried samples had a glass transition temperature (T_g_) of 4.43±3.70°C (*n* = 3) (excluding the edge artifact). Figure 3 shows the variation of the ν-OH band location with temperature in a spin-dried sample. Signal sensitivity limitations of the FTIR technique prevented us from measuring the T_g_ of the desiccated samples containing a monolayer of cells. The phase diagram of a trehalose-water binary system [17] was used to estimate the moisture content of the sample using the T_g_ value measured by FTIR spectroscopy (excluding the edge artifact). The average moisture content of the samples was 0.114±0.087 gH_2_O/gdw. The estimated moisture content is lower than the bulk moisture content of the samples measured by gravimetric analysis (0.214±0.066 gH_2_O/gdw, *n* = 5), since edge artifact has higher moisture content. The bulk moisture content value could be closely approximated from the spatial integration of the water ν-OH peak throughout the sample, which gave a value of 0.195±0.041 gH_2_O/gdw. Bulk moisture contents of the spin-dried solutions that did not contain any cells (used in the FTIR measurements) was not significantly different from those of the spin-dried monolayer of cells (0.215±0.105 gH_2_O/gdw; *n* = 5). Cells remained attached to the surface during spinning and retained their original positions. These results demonstrated that spin-drying produces cell samples that are uniformly dried and highly desiccated.

### Intracellular Trehalose

**Figure 4:**
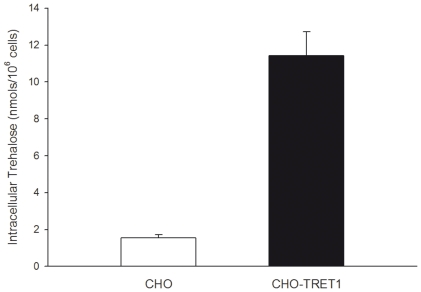
Quantification of intracellular trehalose in wild-type CHO cells and CHO-TRET1 cells. Cells were incubated in fully complemented cell culture medium containing 400 mM trehalose for 4 hours (*n* = 3, ± SD).

HPLC analysis was performed on extracts from both wild-type and TRET1-transfected cells that were pre-incubated with 0.4 M trehalose for 4 h (Figure 4). HPLC analysis indicate that the CHO-TRET1 cells contained an intracellular trehalose concentration of 11.43±1.30 nmol trehalose per million cells (*n* = 3, ± SD), which is about seven-fold higher than wild-type CHO cells under similar conditions (Figure 4). Assuming a spherical cell form with a diameter of about 11 µm (determined by microscopy) and 70% water content, one can calculate the water content of 0.696 µl per million cells [19]. The intracellular concentration of trehalose in the CHO-TRET1 cells can then be estimated as 23.5±2.7 mM. No significant change in morphology of the cells was observed due to the presence of intracellular trehalose in both CHO and CHO-TRET1 cells prior to spin-drying (data not shown).

### Cell Viability after Spin Drying

**Figure 5:**
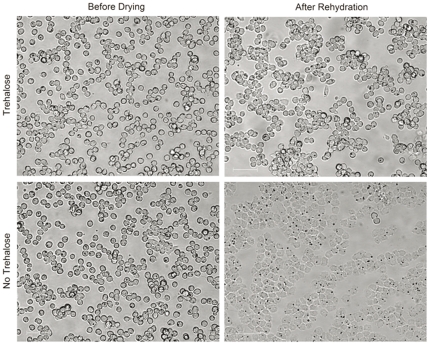
Micrographs of CHO-TRET1 cells before spin-drying and after spin drying and rehydration. Bars in figures indicate 50 µm.

**Figure 6:**
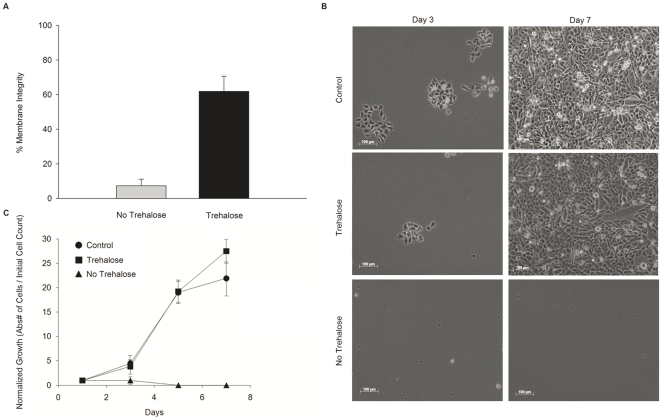
Survival of CHO-TRET1 cells spin-dried in buffers with or without trehalose and rehydrated immediately following desiccation. (A) Membrane integrity of spin-dried cells 45 min after rehydration (B) Micrograph of the cell samples after spin drying and rehydration. (C) Growth of cells after spin-drying and rehydration. The values were normalized to the initial cell count (*n* = 10, ± SD).

Morphology of the CHO-TRET1 cells changed significantly when desiccated in the trehalose-free buffer, whereas cells desiccated in presence of extracellular trehalose were able to preserve the pre-desiccation morphology to a large extent following desiccation and rehydration (Figure 5). Significant changes in morphology of wild-type CHO cells were observed with and without intracellular trehalose following spin-drying, both in presence and absence of trehalose. Post-spin-drying morphology closely resembles CHO-TRET1 cells spin-dried without extracellular trehalose as shown in Figure 5 (data not shown). Survival of spin-dried cells was determined 45 min after rehydration. As shown in Figure 6a, CHO-TRET1 cells pre-loaded with trehalose and spin-dried in the presence of 1.8 M trehalose exhibited 61.9 ± 8.75% (*n* = 10, ± SD) membrane integrity, while cells spin dried without extracellular trehalose had an average membrane integrity of only 7.35±3.79% (*n* = 10, ± SD). Spin-drying CHO-TRET1 cells without intracellular trehalose (both in presence and absence of extracellular trehalose) failed to preserve any membrane integrity. Spin-dried wild-type CHO cells that have undergone the same trehalose-loading protocol as the CHO-TRET1 cells, show an average membrane integrity of 5.14±2.19% (*n* = 3, ± SD) in presence of extracellular trehalose, and 1.12±1.16% (*n* = 3, ± SD) in absence of extracellular trehalose. Spin-drying wild-type CHO cells without intracellular trehalose and extracellular also failed to demonstrate any membrane integrity following rehydration. Figure 6b shows representative micrographs of spin-dried cells following rehydration on days 3 and 7. CHO-TRET1 cells desiccated in presence of extra and intracellular trehalose were able to divide and form colonies, whereas the same cells desiccated without trehalose did not form any colonies (Figure 6b). Cells dried in presence of intra and extracellular trehalose buffer showed growth patterns comparable to control cells (Figure 6c), whereas cells dried under all other conditions failed to demonstrate recovery and long-term growth.

### Cooling and Cryogenic Storage Studies

**Figure 7:**
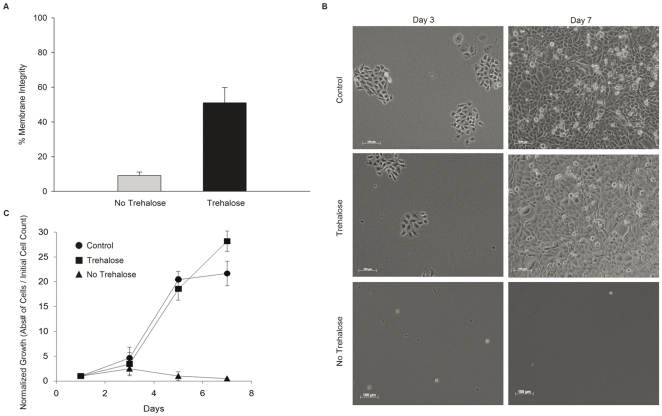
Survival of CHO-TRET1 cells spin-dried in solutions with or without trehalose, then stored in LN_2_ for 1 h, and finally rehydrated. (A) Membrane integrity of spin-dried cells stored in LN_2_ for 1 h and 45 min after thawing and rehydration (B) Micrograph of the spin-dried cells after thawing and rehydration. (C) Growth of spin-dried cells after thawing and rehydration. The values were normalized to the initial cell count (*n* = 10, ± SD).

Spin-dried cells subsequently cooled to −196°C in the presence of trehalose exhibited 51.1±8.3% (*n* = 10, ± SD) membrane integrity, while cells spin dried without extracellular trehalose displayed an average membrane integrity of only 9.1±2.0% (*n* = 10, ± SD), (Figure 7 a, b). Representative micrographs of cells that were desiccated with and without trehalose are shown at days 3 and 7. The micrographs clearly show that spin-dried cells stored in LN_2_ form viable colonies and exhibit growth patterns similar to control cells that were not dried (Figure7c).

A known qualitative way for determining ice crystal formation is the appearance of opacity (or visible ice formation) when solutions are cooled below their freezing point. If there is no observable opacity, the sample is assumed to achieve “apparent” vitrification. Spin-dried samples upon exposure to LN_2_, underwent apparent vitrification. However, the same spin-dried samples were frozen slowly (at 1°C/min) using a controlled rate freezer to −120°C before storing in LN_2_, the samples turned opaque indicating ice-crystal formation and the average viability of the samples was 5.54±4.81% (*n* = 3, ± SD).

## Discussion

We investigated cryogenic storage of highly desiccated mammalian cells in the presence of trehalose. Spin-drying was used to rapidly and uniformly desiccate CHO cells. Highly desiccated spin-dried samples undergo vitrification when cooled rapidly to cryogenic temperatures. Following rewarming and rehydration, ∼51% of the cells that experienced vitrification survived. However, the spin-dried samples that were frozen at a slower rate experienced ice crystallization and displayed a significantly lower level of membrane integrity (∼6%). Unlike the vitrified samples, long-term growth studies of the spin-dried samples cooled to cryogenic temperatures at −1°C/min indicate poor growth patterns (data not shown).

### Moisture Content and Viability of Cells

Recently, spin-drying has been used to create highly desiccated sugar films on a glass substrate [2]. Employing a similar technique, we were able to produce samples that have an estimated average moisture content of <0.12 gH_2_O/gdw. The moisture contents of the samples were estimated from the FTIR measurement of the T_g_ of the spin-dried samples (without cells). As demonstrated gravimetrically, this moisture content does not change appreciably in presence of cells. Recovery of 62% of spin-dried cells at <0.12 gH_2_O/gdw is highly encouraging as this value is lower than the minimum moisture content at which viable cells were recovered using other techniques: A study by Acker et al. [9] did not report any viable cells below a moisture content of ∼0.35 gH_2_O/gdw while Ma et al. [8] reported ∼0.2 gH_2_O/gdw as the limit below which no viable cells were recovered. In both the studies cells were desiccated by evaporative drying after suspension in sessile droplets, and the moisture contents of the samples were measured using bulk gravimetric techniques. Using a faster nebulization based drying technique, Reátegui and Fowler [20] reported some cell viability (<10%) at ∼0.1 gH_2_O/gdw and Guo et al. [21] reported recovery of viable cells following complete desiccation using air-drying. An FTIR-based technique was used to detect the water content of the samples and authors reported no detectable water in the dried samples. However, in a follow-up publication the authors indicated that the moisture content measurement might have been inaccurate [22]. It is likely that the results reported here might represent the lowest moisture levels obtained for uniform desiccation of cells.

### Uniformity of the Spin-Dried Samples

The ability of the spin-drying technique to create uniformly desiccated samples is a major advantage over other cell-drying techniques. Most of the cell-desiccation techniques developed so far have the problem of desiccated samples being highly non-uniform. Desiccating cells using traditional passive drying techniques results in substantial spatial non-uniformity with a water-rich center and a drier peripheral region [11]. Stability of cells following dry-processing depends on the thermodynamic and kinetic factors associated with residual moisture [23], [24]. To successfully preserve cells in a desiccated state it is crucial that an optimum thermodynamic/kinetic state is reached uniformly throughout the sample. Regions with higher residual moisture may have higher chemical reactions thereby increasing chances of degradation of cells. Moreover, such a heterogeneous system continues to evolve during storage causing degradation of the desiccated cells. A high degree of spatial uniformity of moisture content in spin-dried samples may ultimately prove to be beneficial in developing a better dry-processing technique for mammalian cells.

### Pre-Desiccating Cells Prior to Cryogenic Storage

Following spin-drying, storage stability of the desiccated cells was ensured by cryogenic storage at −196°C, which is significantly lower than the T_g_ of the desiccated samples. Storage of desiccated cells at cryogenic temperature is a relatively new approach in cell stabilization. Visual inspection of spin-dried samples stored in LN_2_ indicates apparent vitrification of the samples (data not shown). A review of traditional vitrification protocols indicates that the techniques involve careful and elaborate processing of cells in highly concentrated vitrifying agents having concentrations in the range of 6–8M [1. 25]. The only other study we are aware of storing pre-desiccated samples in LN_2_ is the sperm preservation study by Li et al. [7]. In this study trehalose was introduced into the mouse sperm samples by using α-Hemolysin. The samples were desiccated in a trehalose-EGTA buffer using a forced convection technique in a specialized chamber. The pre-desiccated samples were then stored at cryogenic temperature. Even though the sperm samples were found to be immotile and nonviable upon thawing and rehydration, the nuclear materials were found to be potent and were successfully used to fertilize ova using ICSE technique. Here we have demonstrated the ability of mammalian cells to retain their viability and growth potential upon uniform desiccation to very low moisture level followed by storage at cryogenic temperature.

### Trehalose Loading Using TRET1 Trehalose Transporter

During spin-drying trehalose was present on both sides of the cell membrane to prevent fusion and subsequent loss of membrane integrity [26]. Trehalose was loaded into the cells using the TRET1 trehalose transporter, derived from the anhydrobiotic African chironomid *Polypedilum vanderplanki*. The larvae of *P. vanderplanki* are the largest known multicellular organism that can withstand almost complete dehydration without any detectable damaging effects for as long as 17 years [27]. TRET1-transfection greatly improved loading of trehalose into CHO cells compared to control (non-transfected) cells (Figure 4). Ma et al. [8] reported values of intracellular trehalose of up to 100 mM by fluid phase endocytosis in human embryonic kidney cells. The difference in uptake are likely to be cell-line specific since CHO cells only accumulated 3.17 mM trehalose after incubation in 400 mM trehalose solution for 4 hours. However, CHO-TRET1 cells did not reach equilibrium condition with the extracellular concentration after 4 hours. This might be to due to sub-optimal expression level of TRET1 transporter from *P. vanderplanki* in mammalian cells. We are currently in the process to increase expression levels of TRET1 by codon optimization. Membrane integrity of CHO-TRET1 cells was over 60% after spin drying and immediate rehydration (Figure 6a). Growth data demonstrates that CHO-TRET1 cells desiccated in buffer containing trehalose have virtually the same growth characteristics as the control cells that did not undergo drying (Figure 6c). A similar trend is observed following the storage of desiccated cells in LN_2_ (Figure 7). These results reconfirm the protective effect of trehalose during desiccation.

### Strategies for Non-Cryogenic Storage

FTIR studies of the spin-dried samples indicate that the T_g_ of the spin-dried samples is ∼4.4°C, which would suggest that at ambient temperature, spin-dried samples do not form a stable glassy environment as required for storage. In fact, studies with lyophilized pharmaceutical compounds have shown that molecular motion might occur even in the glassy state [28], [29] leading to potential instabilities in desiccated cells during storage. The stable long-term storage of cells is thus believed to require storage temperature 30–50K below the T_g_. One strategy to increase the T_g_ of the spin-dried samples is to reduce moisture content of the samples by increasing the spinning speed. However, an alternative approach may be to add specific molecules to the drying solution, which will increase the T_g_ at a given moisture content. It is known that by adding dextran to the trehalose solution, the moisture content of trehalose-dextran glasses can be significantly increased above that of trehalose glasses [12]. Similar results can be obtained by cross-linking trehalose with borate ions [30]. Additionally, MacFarlane et al. [31] have shown that a number of salts and sugars can produce high T_g_ in carbohydrate systems, by forming a fully reversibly coordination polymers upon dehydration of dilute solutions. Such approaches might be necessary to achieve stable storage of cells at ambient temperatures. However, storage of cells at ambient temperatures was beyond the scope of this study.

### Conclusion

In conclusion, the pre-desiccation of cells prior to vitrification at cryogenic temperature is an exciting approach in cryopreservation. We have demonstrated that spin-drying is an excellent technique to rapidly and uniformly desiccate cells to very low moisture contents. It is likely that the results reported here represent the lowest moisture levels obtained for uniformly desiccated cells. Spin-dried cells were stored at cryogenic temperatures without using exceedingly high concentrations of CPAs and complex CPA loading protocols. At present we are investigating ways to stabilize cells at temperatures higher than cryogenic temperature. Successful storage of cells at temperatures higher than cryogenic temperature can address some of the long-standing bottlenecks in ambient temperature storage of mammalian cells.
